# Effect of a Nutritional Intervention in Athlete’s Body Composition, Eating Behaviour and Nutritional Knowledge: A Comparison between Adults and Adolescents

**DOI:** 10.3390/nu8090535

**Published:** 2016-09-07

**Authors:** Marcus Nascimento, Danielle Silva, Sandra Ribeiro, Marco Nunes, Marcos Almeida, Raquel Mendes-Netto

**Affiliations:** 1 Department of Nutrition, Federal University of Sergipe, São Cristóvão 49100-000, Brazil; marcusnascimentone@gmail.com (M.N.); daniellegoes@ufs.br (D.S.); 2 School of Public Health, University of São Paulo, São Paulo 01246-904, Brazil; smlribeiro@usp.br; 3 Department of Medicine, Federal University of Sergipe, São Cristóvão 49100-000, Brazil; nunes.ma@ufs.br; 4 Department of Physical Education, Federal University of Sergipe, São Cristóvão 49100-000, Brazil; mb.almeida@gmail.com

**Keywords:** body composition, nutritional intervention, athletes, eating behaviour

## Abstract

The objective of the present study is to evaluate and compare the effect of a nutritional intervention between adolescent and adult. In a before and after quasi-experimental clinical study, 32 athletes (21 adults, age range 20–32 years; 11 adolescents, age range: 12–19 years) participated in a nutritional counselling consisting of four consultations separated by an interval of 45 to 60 days. The athlete’s eating behaviour, body composition and nutrition knowledge were evaluated at the beginning and at the end of the protocol. Both groups increased lean body mass and nutritional knowledge. Adolescents increased their mid-arm muscle circumference and improved meal frequency, and daily water intake. Athletes of both groups improved their ingestion of vegetables and fruits and decreased the ingestion of sweets and oils. Adolescents showed a higher prevalence of individuals that remained within or approached to the recommendations of sweets. This is the first study to evaluate and compare the effect of a nutritional intervention between adolescent and adult athletes body composition, eating behaviour and nutritional knowledge. The nutritional counselling has been effective in promoting beneficial changes on the athlete’s eating behaviour, nutritional knowledge and body composition, however, some healthy changes were only experienced by adolescents, especially in the frequency of meals and the intake of sweets.

## 1. Introduction

A balanced diet is important for an improved sports performance and for health. During exercise, athletes may suffer from the depletion of glycogen stores, dehydration and muscle damage. Thus, the ingestion of nutrient rich foods (lean meat/milk, fruits, vegetables and complex carbohydrates) and water may improve thermoregulation, enhance energy stores, maximize muscle protein synthesis and provide the supply of vitamins and minerals [1].

Although the importance of adequate nutrition has been well established [1], many athletes have shown several nutritional inadequacies [2,3,4,5]. Some authors have suggested that the dietary errors found in an athletic population may be due to low levels of nutritional knowledge and a lack of adequate nutritional counselling [6].

One strategy to improve the nutrition knowledge of athletes and coaches could be tailored nutrition programs. In the 1990s, some universities settled upon nutritional educational programs linked to their own sports department [7,8]. More recently, other nutritional interventions have also been developed [9,10]. However, these programs did not have their effectiveness evaluated, and they reflect a different reality from which most athletes are exposed, as they have extensive protocols and are dependent on a multi-disciplinary team.

There are few published studies involving nutritional interventions in athletes, and due to the different methodologies used, the results are inconsistent. Collison [11] did not find changes in the dietary intake of athletes, after participating in two nutritional workshops. In contrast, Carmo, Marins and Peluzio [12] observed a significant reduction in the dietary fat intake and the body fat percentage in Jiu-Jitsu athletes, after nine months of nutritional counselling.

Adolescent athletes are at a high nutritional risk because of the high energy cost of training. In addition, a number of nutrients are needed for the processes of growth and development. Proteins are needed to maximize muscle protein synthesis, calcium and vitamin D are important in the development and maintenance of skeleton, essential fatty acids may provide energy to support the growth and maturation, iron would prevent adverse athletic performance due to suboptimal iron stores, and so forth [1]. Nonetheless, few studies have studied nutritional interventions in this type of population, and most of them have only focused on improving the athlete’s hydration practices [13,14] or their nutritional knowledge [15]. It is necessary to investigate this type of program in order to develop specific strategies to these individuals.

In this context, the objective of the present study is to evaluate and compare the effect of a nutritional intervention in athlete’s body composition, eating behaviour and nutritional knowledge. Our secondary aim is to compare the effect of the nutritional intervention between adult and adolescent athletes.

## 2. Materials and Methods

This study was conducted according to the guidelines laid down in the declaration of Helsinki and all procedures involving human subjects were approved by the Research Ethics Committee of the University Hospital UFS (CAAE 08574213.4.0000.5546).

The work was conducted with athletes from a Brazilian program for athlete support, the “Bolsa Atleta”, in the city of Aracaju, Brazil. This program provides financial aid to featured athletes who compete in the Olympic, Paralympic, and non-Olympic sports.

Data regarding the number of athletes of the program were provided by the SEJESP (Department of Youth and Sports in the city of Aracaju, Brazil). The program includes different athletes yearly, based on their sports results (state competitions). At the time of this study, 80 athletes were enrolled, from which five were in the gold category (international competitions), 25 in the silver category (national competitions), and 50 in the bronze category. After checking the inclusion and exclusion criteria, the eligible athletes were invited. These athletes were invited to take part of the study.

The inclusion criterion was based upon being a beneficiary of the program. There were no age or gender restrictions. The exclusion criteria were being in any concomitant nutritional counselling programs, or having any disease or health condition that required a specialized dietary planning.

### 2.1. Study Design

The work consisted of a quasi-experimental clinical trial with a pre and post design. The data collection took place from February 2012 to March 2014.

Written informed consent was obtained from all subjects. In the case of the adolescents, the consent form was sent to their respective responsible parents or guardians.

**Figure 1:**
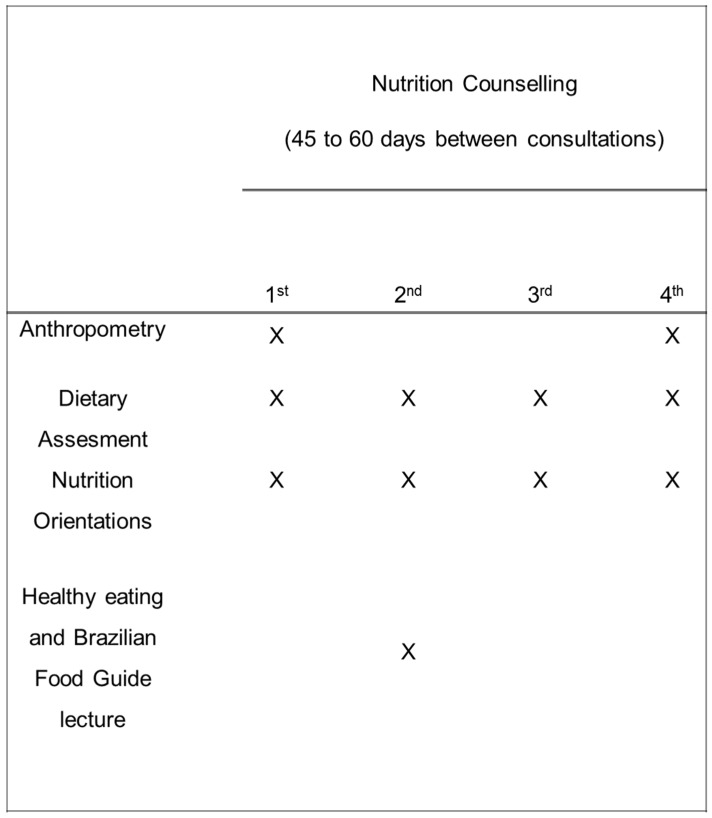
Experimental design of the study.

The program consisted of four visits with nutritional counselling and one lecture related to Brazilian Food Guide. [16,17]. During the intervention period, dietary and anthropometric measurements were performed. The data obtained before (first visit) and after the nutritional intervention (fourth visit) were compared. Figure 1 shows the experimental design of the study.

### 2.2. Anthropometric Evaluation

The anthropometric measures were performed following the techniques proposed by Lohman et al. [18]. Height was measured to the nearest 0.1 cm using a stadiometer (Altura Exata^®^, Altura Exata, Belo Horizonte, Brazil and body weight was measured to the nearest 0.1 kg using an electronic scale (P150M^®^, LÍDER, Araçatuba, Brazil). The mid-arm circumference was measured to the nearest 0.1 cm using a flexible and non-elastic tape (Sanny^®^, Sanny, São Bernardo do Campo, Brazil). All measurements were performed while the subjects wore no shoes and only light clothes.

Using a Lange Skinfold Calliper, the following skinfold thickness measurements were taken: triceps, subscapular, suprailiac, abdomen, thigh, axilla, and chest. These were measured to the nearest 0.1 mm, with the average of three measurements at each site being used for analysis. Evans et al. [19] equation was used for determining body fat percentages in both male and female adult athletes. In the adolescents, the percentage of body fat was estimated by Lohman’s equation [20]. The triceps skinfold and the mid-arm muscle circumference were used to calculate the mid-arm muscle circumference (MAMC) in both groups [21].

### 2.3. Dietary Intake Assesment

The dietary intake was assessed by a sports nutritionist using a 24-h food recall. This method consisted of a written or verbal report about the food intake during the previous 24 h. The data on the food currently consumed, weight information, portion sizes, and food preparation techniques, were also collected.

A photo album was used as a resource to assist the respondents in remembering the food portions consumed, and thereby increasing the reliability of the information provided. This album consisted of utensils and food designs in three normal sizes (small, medium, and large) [22,23].

The energy content of each athlete’s food intake was calculated using the Nutrition Data System for Research Software (NDSR) Version 2011 (NCC, Minneapolis, MN, USA) The daily water intake included water from food and beverages. The water consumption during training was based on the water from beverages used. Soft drinks, tea, or coffee, were not included in the water analysis.

The food servings were compared with the recommendations proposed by the Brazilian Food Pyramid [16,17]. As athletes might have different nutritional inadequacies, which may influence the nutrition advices given, we decided to analyse the nutrition intervention effects on food portions by grouping athletes according to their classification in adequate, low or high consumers of each food group [16,17]. The prevalence of the individuals who approached or remained within the recommendations of the protocol was also analysed.

The interval between meals was calculated from the mean interval between each meal. The characterisation of each meal was defined based on Burke et al. [4]. Breakfast was regarded as the first meal of the day between 05:00 and 10:00, the morning snack as the meal between 10:00 and 11:59, lunch as the meal between 12:00 and 14:59, the afternoon snack as the meal between 15:00 and 17:59, dinner as the meal between 18:00 and 20:59, and supper between 21:00 and 04:59.

Any food or energy containing drink consumed within a 30-min period was considered a “meal”. The morning snack, the afternoon snack, and supper, were grouped into a single category called “snacks”, while breakfast, lunch, and dinner, were considered to be “main meals”. The prevalence of meal omission was also calculated. Furthermore, the time adequacy of pre and post-training meals were analysed according to the recommendations proposed by Aragon and Shoenfeld [24], where the interval between the pre-training and the post-training meals should be of three to four hours.

### 2.4. Nutritional Intervention

The nutritional intervention was divided into four face-to-face consultations, lasting for 45 to 60 min (Figure 1). The nutritional advice was given by only one sports nutritionist in order to minimize bias. Training routines, diet, anthropometric measurements, and personal data, were collected during the first meeting. From the initial analysis of eating habits and athlete’s routine, specific dietary counselling was given and goals were set to improve diet quality. To increase the athlete’s adherence, three days of the week were made available for consultations. At the end of the meetings, or by telephone, the athletes were scheduled for revaluations, and these occurred in the range of 45 to 60 days after the previous evaluation.

During the intervention, the athletes individually participated in a nutritional educational lecture about the Brazilian Food Guide [16,17] (2nd meeting). The participants were presented and clarified about the principles of healthy eating, focusing on the importance of each food group. The educational protocol aimed to improve the nutritional knowledge and to motivate the adoption of dietary practices that would promote health and athletic performance.

**Figure 2:**
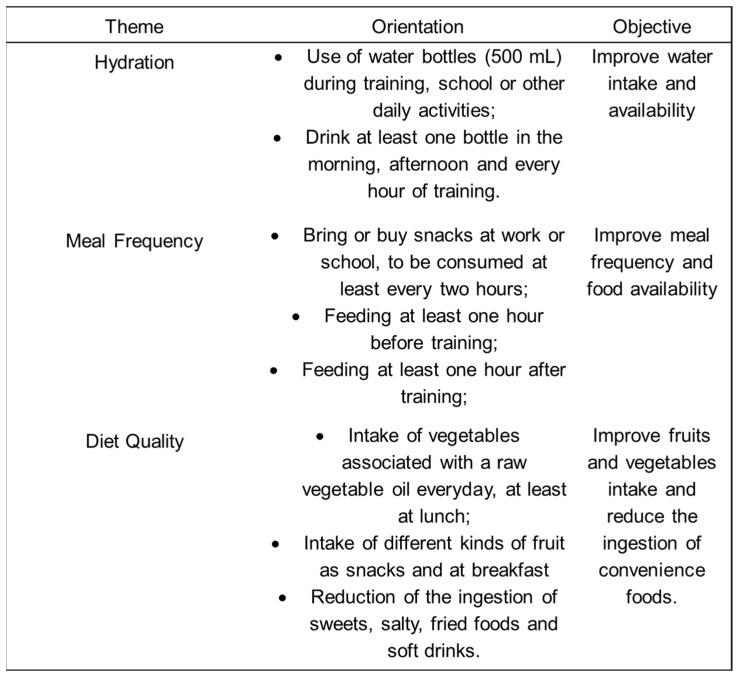
Issues addressed in the consultations.

Adherence to guidelines was verified at each follow-up evaluation, as well as the dietary adjustments, in accordance with the current objectives of training and competition. At the end of each visit, the athletes received a list of specific nutritional advice. In addition, the aspects described in Figure 2 were reinforced during all meetings. To maintain the athlete’s motivation, a group was created on a social network, whereby all participants received information about healthy eating tips and recipes. The information was posted monthly by the sports nutritionist and included advice about the preparation of pre and post-training meals, healthy hydration practices during training and competitions and other sport nutrition issues.

### 2.5. Nutritional Knowledge

A nutritional knowledge test based on the studies of Gonçalves [25] and Zawila, Steib and Hoogenboom [26] was applied. The questionnaire had 14 questions divided into three sections. The first section contained three multi-choice questions about the basic aspects of nutrition. The second part consisted of a question related to the Brazilian Food Guide Pyramid, where the athletes had to fill in the pyramid with the correct food groups. The third section addressed the issue of sports nutrition and was comprised of a matter containing 10 statements to which the athletes should mark “yes” if they agreed with the statement, “no” if they disagreed with the statement, or “do not know” if they were unsure. The correct issues were worth a plus point and the wrong or “do not know” answers received no points. The average percentage of correct answers was calculated and they were compared between the groups before and after the intervention.

The questionnaire had its discriminative validity determined in a previous study by our research group [27]. The test was applied to 19 graduates of the 4th period of nutrition and to 16 adolescent athletes. To be considered valid, the questionnaire should be able to differentiate the participants at different levels of knowledge. After the application, the students had a significantly higher mean percentage of correct answers (97.4%) than did the athletes (57%).

### 2.6. Statistical Analysis

The statistical analysis was performed using SPSS Software Version 17.0 (SPSS Inc., Chicago, IL, USA). The data normality was verified by the Kolmorgorov–Smirnov test. Normally distributed data were presented as a mean and standard error (SE), while non-normally distributed variables were log-transformed before statistical analyses to avoid skewed data and are presented as geometric means and back-transformed 95% confidence intervals (95% CI) [28].

Student’s *t*-tests and Pearson’s chi-square test were used to access whether any demographic, anthropometric or dietary measures where different between groups at baseline. The significance of within-group changes in numeric variables (within-group analyses) was determined using paired *t*-tests. The categorical data was compared over time using McNemar’s Test. Since there were baseline differences between groups with respect to anthropometric measures, number of meals, interval between meals, daily water intake and water intake during training, a general linear model univariate analysis (ANCOVA) was used to determine whether the change scores of these variables (post-pre) where different between adolescents and adults, after adjusting for pre-intervention values.

The internal consistency of the nutritional knowledge questionnaire was obtained by the Cronbach’s alpha coefficient (α). This coefficient ranges between 0.00 (no reliability) to 1.00 (perfect reliability). The minimum value of 0.70 was recommended by Rowland, Arkkelin and Crisler [29]. Statistical analyses of the intervention effects on nutrition knowledge were carried out using a two factor (group and time) analysis of variance (ANOVA). For all analysis, a statistical significance was set at *p* < 0.05.

## 3. Results

**Figure 3:**
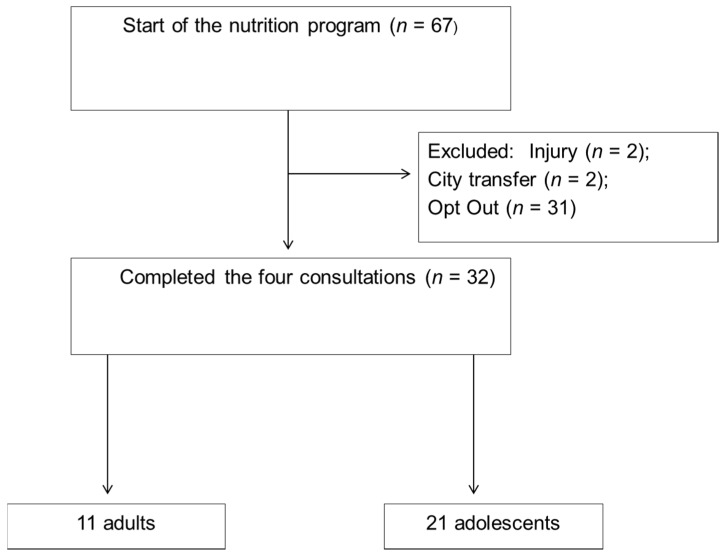
Study diagram.

After checking the inclusion and exclusion criteria, 67 athletes were eligible to participate in the research. Of these, only 32 athletes completed the four-consultation protocol. The reasons for attrition, as well as the description of the final sample are at Figure 3. The participants were 32 athletes of the following sports: fighting (boxing, taekwondo, karate, judo, jiu-jitsu, capoeira, and wrestling, *n* = 16), athletics (*n* = 3), cycling (*n* = 1), swimming (*n* = 6), tennis (*n* = 2), beach volleyball (*n* = 1), surfing (*n* = 1), rowing (*n* = 1) and sailing (*n* = 1). The sample consisted of 21 adolescents (65.6%, age range: 12–19 years) and 11 adults (34.4%, age range: 20–32 years), with a mean age of 15.4 years (SE: 0.35) and 23.7 years (SE: 0.53), respectively. All of the adults were male, while six adolescents (28.6%) were female and 15 were male (71.4%). There was no difference in the results when they were analysed without the female athletes; thus, they were included. The adolescents and the adults had an average of 12.8 (SE: 1) h and 16.2 (SE: 1.2) h of training per week, respectively.

Most of the athletes in both groups (95.2% of adolescents and 81.8% of adults) had a goal of maintaining or gaining lean mass. Only one adolescent (4.8%) and two adults (18.2%) had the intention of reducing body mass.

Table 1 shows the anthropometric and body composition values of the athletes before and after nutritional counselling. Both groups increased their body mass and lean body mass (kg), however, only the adolescents increased MAMC. There were no differences in the changes between the groups (ANCOVA, *p* > 0.05).

**Figure 4:**
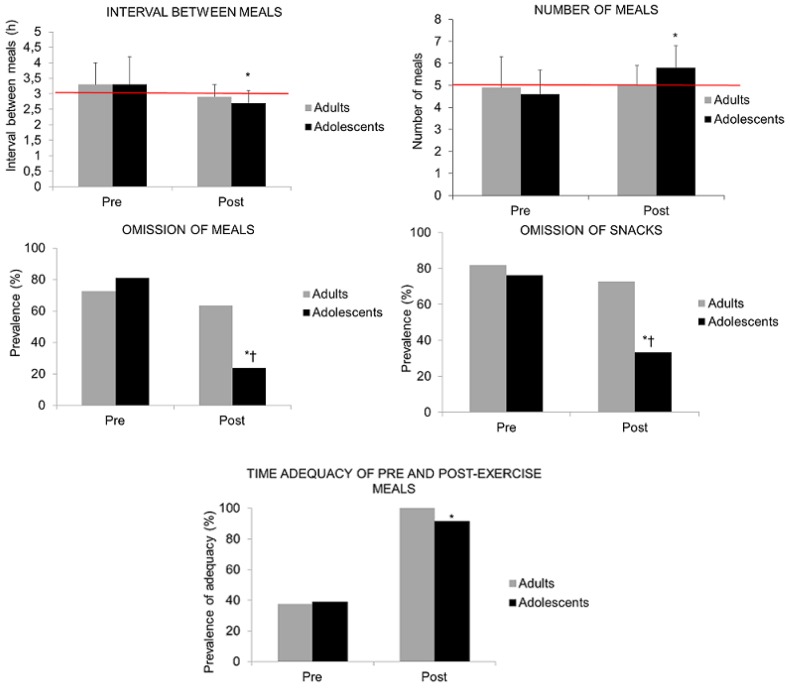
Number of meals, interval between meals, meal omission, snack omission, and time adequacy of pre and post-training meals, before and after nutritional counselling. The red lines indicate the recommendations of at least five meals a day (number of meals) and a maximum of three hours between meals (interval between meals). * *p* < 0.05, pre versus post. ^†^ *p* < 0.05, adults versus adolescents.

The analysis showed an increase in the number of meals for young athletes, as well as a significant reduction in the interval between the meals; however, there were no group effects on the changes in these variables (ANCOVA, *p* > 0.05) (Figure 4). The adolescents also showed a significant reduction of meal and snack omissions. Both groups increased the time adequacy of pre-training and post-training meals (Figure 4). As all of the adults were suited to the recommendations, it was not possible to apply within-group inferences.

The within-group analysis showed that there was a statistically significant increase in daily water intake among the adolescents (Table 2). There were no differences in the change scores between the groups (ANCOVA, *p* > 0.05).

Table 3 shows the athletes ingestion of food portions according to their baseline classification. Participants with low intake of legumes and vegetables increased their ingestion. Athletes that demonstrated high intakes of meat and eggs, sweets and oils decreased their ingestion after the intervention. We also found a high prevalence (more than 50%) of individuals that remained within or approached to the recommendations of cereals, fruits, vegetables, meat and eggs, and oils and fats. When these values were compared between groups, the adolescents showed a higher prevalence of individuals that remained within or approached to the recommendations of sweets (Adolescents: 71.4%, adults: 18%).

The nutrition knowledge questionnaire internal consistency value was obtained through Cronbach’s coefficient. These values showed an acceptable reliability for the adults (0.84) and the adolescents (0.81). Both groups had an increment in total and food pyramidal nutritional knowledge (Table 4).

## 4. Discussion

To our knowledge, this is the first study to evaluate and compare the effect of a nutritional intervention between adolescent and adult athletes. The results have shown that both groups improved their body composition, their dietary intake and nutrition knowledge, however, the adolescent had a higher improvement on body composition, meal frequency and sweets intake than adults.

### 4.1. Body Composition

After about eight months of nutritional counselling, the adolescent athletes increased their MAMC, while and showed a trend towards significance to increase their lean mass (*p* = 0.051). Since the results were consistent with the objectives outlined in the consultations, the specific nutritional advice that was given may have contributed to the changes in their body composition (most of the athletes reported that lean mass gain or maintenance as a goal).

It should be noted that adults had a higher increase in fat than adolescents (ANCOVA, *p* < 0.05). An increase in body fat may occur during nutrition interventions focusing on body mass gain [30]. However, due to a more anabolic profile [31], the adolescent athletes may have had a greater capacity to gain muscle mass than the adults, without changes in fat mass.

Other nutritional intervention studies have shown significant changes in an athlete’s body composition and also had their planning directed to their goal. Garthe et al. [30] supported a total of 21 athletes for at least eight weeks who aimed to gain body mass. The participants received nutritional counselling by two nutritionists and at the end of the study they showed an increase in their body mass and their lean body mass (approximately 1.7 kg). More recently, Carmo, Marins and Peluzio [12] found a significant reduction in body mass and body fat percentage in 20 Jiu-Jitsu athletes after participating in a specific nutritional intervention for reducing body mass.

These results are of great relevance, as athletes may have difficulty in achieving the desired body shape, and they tend to adopt inappropriate strategies which can be harmful to health and sports performance [11]. In these situations, nutritional counselling should be indicated as a strategy to promote changes with a greater efficiency and quality.

### 4.2. Meal Frequency

The division of the total caloric intake in frequent meals (with a three-hour interval) can be beneficial for athletes, since it reduces the risk of gastrointestinal distress and provides a greater flexibility in the amount and the variety of food to be ingested. These factors may help to improve diet quality and nutrient distribution throughout the day [3]. However, both age groups of athletes showed a high prevalence of meal omission, mainly of snacks, which contributed to the inadequacy in the number of meals and the interval between them.

The increasing availability of healthy foods is seen as a facilitator of eating behaviour changes, especially among adolescents, since they are exposed to foods of a low nutritional value and a high energy density, especially in school [32]. Thus, during these consultations, the athletes received instructions regarding the preparation of practical snacks with a high nutritional content, which should be consumed at home, work, or at school, in order to increase healthy food accessibility and meal frequency. These guidelines were also reinforced by their social network, where they received tips on examples of healthy meals.

After the nutritional intervention, the adolescents increased their number of meals, reduced the interval between them, as well as an omission of snacks. In addition, both of the groups increased their time adequacy of pre and post-training meals.

Despite the scarcity of studies about athletes that perceive barriers for healthy eating, the literature suggests that they may have difficulty in maintaining an adequate frequency of meals, due to the exhausting routine caused by a high work load of training and associated with other tasks (e.g., work and school) [33]. An anamnesis taken showed that all of the adolescents attended school lessons in the morning and sports training in the afternoon, or at night, while the adults, in addition to training and studying, had much of the day filled with working hours. Studies that have focused on the analysis of adults have perceived barriers to adopt a healthy eating habit, observed that the most cited reason is a lack of time for the preparation and the consumption of food [34]. Thus, our hypothesis is that by having a greater number of obligations than adolescents (family, education, and employment), the adults have found a greater difficulty in feeding, especially between “main meals”, despite having the same hours of training.

### 4.3. Water Intake

Despite of the importance athlete’s hydration behaviour, to our knowledge, only two studies have analysed the effect of nutritional interventions on athlete’s hydration practices. Kavouras et al. [14] and Cleary et al. [13] have improved the hydration status in young athletes, by individual prescriptions, and have increased the accessibility of this nutrient, respectively.

In the present study, the participants were advised to drink water from 500 mL bottles at different times of the day. These strategies could facilitate quantification and the perception of water intake, as well as improving its availability. After nutritional counselling, the adolescents increased their daily water intake. For both groups, although their improvement in water intake during training was not statistically significant, the result was clinically relevant, since they doubled their ingestion.

### 4.4. Food Portions

There are several methods to evaluate dietary intake. The average intake of a nutrient, or the prevalence of individuals facing a guideline are the most used, however, some considerations need to be analysed when performing nutritional intervention studies. The literature suggests that small progressive changes in a diet are more effective and sustainable than big ones [35]. The time necessary for an eating behaviour change may vary depending on the social and environmental factors specific to each individual. Thus, one must expect an individual to pass from an intake category of “inadequate” to “adequate”. This may be a conservative assessment (e.g., not eat any fruit portion and begin to consume four servings), preventing the detection of small changes. Some studies have used an average as the evaluation method. However, as athletes can have different types of food inadequacies within the same sample, the average intake of a nutrient may include athletes who have an inadequate intake and those who are adequate. This grouping can lead to a bias in the results interpretation, since those with an intake within the recommendations, were oriented to maintain it, contributing to the average intake unchanging after the intervention. Thus, to reduce this bias, athletes were grouped according to their baseline classification of food portions ingestion.

After the intervention, athletes classified as low consumers of fruits, vegetables, dairy and high consumers of sweets, meat and fats and oils approached to the recommendations of the Brazilian Food Pyramid, which could be considered a positive effect of the nutrition intervention. Data analysis also showed that athletes maintained their adequate intake of most of the food portions. However, participants appeared to have had more difficulties in maintaining the adequacy of sweets, fat and oils and vegetables. Considering that these food habits might take a longer time to change, dieticians should carefully monitor the ingestion of these food portions during nutrition interventions. As athletes are exposed to numerous barriers that preclude a balanced diet, and even for those guidelines that are being met, the strategies have been tightened at each visit. Thus, the maintenance of an adequate intake could also be considered a positive effect.

Both of the groups showed a high prevalence of individuals that approached to or remained adequate within the recommendations, however, when analysing the ingestion of sweets, this prevalence was higher among adolescents. The preference for a sweet taste has been identified in studies involving both adolescents and adults and has been considered a barrier to the ingestion of other food groups [36,37]. In this study, as all athletes were residential, it is possible that the presence of parents in the adolescent consultations may have aided the adherence to the nutritional advices. This would be especially evident with regard to food intake and frequency, as parents were responsible for the courses preparation and its organisation, and thus would provide a greater support for the athletes. Iglezias-Gutiérrez et al. [38] observed that food preferences might not influence an adolescent athletes dietary intake. This might be due to the influence of the family environment on the purchase and selection of meals, which may reduce the chances of ingesting foods that were considered “preferred”.

### 4.5. Nutritional Knowledge

Both groups had an increase in their nutritional knowledge, especially with regard to the topics that related to the Brazilian Food Guide Pyramid. This finding is of a great importance, as athletes receive nutritional information from various sources, mainly from coaches and trainers, who have shown a lack of nutritional knowledge. In addition, unreliable information sources, such as the Internet, magazines, friends, relatives, and media, are widely used for information [6].

In addition to the dissemination of nutritional information topics, specific orientations to the needs and difficulties of each athlete were provided in the present study by a sports nutritionist, which has been considered the most qualified professional to give nutritional advices to athletes. This approach may have been responsible for the better results being found in relation to the researchers that have only used nutritional educational strategies, such as seminars and lectures [11].

Despite the fact that the nutritional intervention strategy has promoted beneficial changes in body composition, dietary intake, and athlete’s nutritional knowledge, it is worth noting that the participants had a low adherence to the protocol adopted. Only 50% of the participants who started the protocol finished the four consultations. Due to the high number of bookings for each athlete, we hypothesized that they had difficulties in making time for the consultations during their daily routine. Future research should focus on the main barriers faced by athletes to adopt healthy eating, and factors such as boredom and tedious teachings may influence the adherence to different types of nutritional intervention. As family and coaches may possibly influence athlete’s food habits, the research protocols should also include these particular populations.

### 4.6. Practical Applications

When consulting athletes it is important for nutritionists to take into account the time for meal preparation, as well as its possibility of storage time, especially in the case of snacks. A minimum of five meals/day is recommended, however, these meals should be gradually inserted to facilitate an athlete’s adaptation, especially in the pre- and post-training period.

The development of practical strategies to increase water availability might be useful, especially in places where it is done through drinking fountains or sports that are practiced in open spaces such as beaches and fields. Even with the existence of general hydration recommendations (500 mL/h of training), it is important, to first of all, respect an athlete's tolerance to the prescribed amount of liquid. The prescription of high-water-content food (e.g., Fruits) may also enhance hydration during the day.

### 4.7. Limitations

Despite the relevance of the results of this study, some methodological limitations must be taken into consideration. The analysis of food intake using a single 24-h recall is a limiting factor on the basis of the intra-individual variability provided by the instrument. However, it was necessary the use of this method due to the operational difficulty in accessing the same participant more than one time, as the athletes trained in different places and had to move to the place of data collection. According to Magkos and Yannankolia [39], the use of a single 24-h recall might be an alternative when you cannot use the instrument more than one time. Other works also used this method [40,41].

## 5. Conclusions

The present study has shown that the nutritional intervention was effective in promoting beneficial changes in athletes’ body composition, eating behaviour, and nutritional knowledge. However, some healthy changes were only experienced by adolescents, especially in the frequency of meals and the intake of sweets.

## Figures and Tables

**Table 1:** Mean (SE) of athlete’s anthropometry and body composition.

Variables	Group	Intervention (*n* = 32)		ANCOVA ^1^
		Pre	Post	*p*-Value ^1^
Body mass (kg)	Adults	69.2 (2.0)	71.4 (2.1) ^3^	0.06
	Adolescents	56.1 (2.2)	57.6 (2.0) ^3^	
BMI (kg/m^2^)	Adults	24.8 (1.3)	25.1 (1.2)	0.056
	Adolescents	20 (1)	20.3 (0.8)	
MAMC	Adults	26.8 (11)	26.9 (12)	0.21
	Adolescents	22.4 (0.6)	23.8 (0.8) ^3^	
ƩSKF ^2^	Adults	80.4 (9)	93.1 (13)	0.57
	Adolescents	20.6 (2)	20.3 (1.4)	
Fat mass (%)	Adults	12.6 (1.4)	14.2 (1.5)	0.58
	Adolescents	14 (1.5)	13.7 (1.1)	
Lean mass (kg)	Adults	60 (1.7)	61.1 (1.6)	0.03
	Adolescents	48 (1.8)	49.2 (1.6)	
Fat (kg)	Adults	8.9 (1.1)	10.3 (1.3)	0.001
	Adolescents	8 (0.8)	8.4 (0.6)	

^1^ *p*-values refer to differences between groups, using ANCOVA on the changes, adjusting for baseline values; ^2^ Adolescents: sum of two skinfold, adults: sum of seven skinfolds; ^3^ *p* < 0.05, pre versus post.

**Table 2:** Geometric mean (95% CI) of daily water intake and water ingestion during training.

Variables	Group	Intervention (*n* = 32)		ANCOVA
		Pre	Post	*p*-Value ^1^
		Mean (95% CI)	Mean (95% CI)	
Daily Water (L)	Adults	4.8 (2.3–9)	5 (2.4–10)	0.30
	Adolescents	3.3 (2–5.6)	3.6 (2.1–6) ^3^	
Water during training (mL/h) ^2^	Adults	233 (5–1107)	576 (63–5268)	0.44
	Adolescents	192 (17–2101)	417 (175–993)	

^1^ *p*-values refer to differences between groups, using ANCOVA on the changes, adjusting for baseline values; ^2^ *n* = 21; ^3^ *p* < 0.05, pre versus post.

**Table 3:** Intake of food portions before and after the intervention.

Portions	Portion Intakes Classification	Age Group		Intervention ^1^ (*n* = 32)		Guidelines ^2^
		Adult *n* (%)	Adolescents *n* (%)	Pre	Post	
Cereals	Adequate	7(50)	7(50)	9.8 (6.7–14)	6.1 (3.5–10) ^3^	6–9
	Low	4(22.2)	14(77.8)	3(1.7–5.5)	3.8 (2.1–6.8)	
Fruits	Adequate	8 (34.8)	15(65.2)	6.6 (5–8.7)	4.8 (2.6–8.6) ^3^	3–5
	Low	3(33.3)	6(66.7)	2.4 (1.2–5)	4.6 (1.6–12) ^3^	
Vegetables	Adequate	2(34.6)	4(65.4)	6.3 (2.5–16)	2.5 (1.4–4.8) ^3^	3–5
	Low	9 (34.6)	17 (66.7)	1.6 (1.6–3.1)	2.2 (0.8–7.8) ^3^	
Meats and Eggs	Adequate	4 (25)	12 (75)	2.1 (1.6–3)	2.8 (1.8–4)	1–2
	High	7 (43.8)	9 (56.3)	4 (3–5)	2.8 (1.7–4) ^3^	
Dairy	Adequate	3 (23.1)	10 (76.9)	5 (3.8–6.8)	3.3 (1.4–8)	3
	Low	8 (42.1)	11 (57.9)	1.8 (1.1–2.9)	2.5 (1.7–3.7) ^3^	
Beans and nuts	High	8 (34.8)	15 (65.2)	4 (2.8–6)	2.6 (1.2–5.5) ^3^	1
	Adequate	3 (33.3)	6 (66.7)	1.2 (0.8–1.8)	2.8 (0.2–6.4) ^3^	
Fats and Oils	Adequate	4 (21.1)	15 (78.9)	2(1.6–2.6)	2.7(1.6–4.7) ^3^	1–2
	High	7 (53.8)	6 (46.2)	4.8 (3.3–7)	2.5 (1.5–4) ^3^	
Sweets	Adequate	9 (45)	11 (55)	2 (1.4–2.8)	3.4 (1.9–6) ^3^	1–2
	High	2 (16.7)	10 (83.3)	8.4 (6–12)	2.9 (1.4–6) ^3^	

^1^ Data expressed as geometric means (95% CI); ^2^ Phillip (1999); ^3^ *p* < 0.05, pre versus post.

**Table 4:** Mean (SE) of athlete’s nutritional knowledge before and after the intervention.

Nutrition Knowledge Categories	Group	Intervention (*n* = 32)		ANOVA (*p*-Value)		
		Before	After	Group	Time	Group × Time
Total	Adults	70 (9)	89 (10) ^1^	0.75	<0.001	0.47
	Adolescents	73.6 (15)	84.6 (11) ^1^			
Basic Nutrition	Adults	89.7 (23)	92 (18)	0.94	0.42	0.77
	Adolescents	92 (12)	97 (13)			
Food Pyramid	Adults	28.4 (26)	77 (14) ^1^	0.85	0.001	0.56
	Adolescents	37 (28)	52 (25) ^1^			
Sports Nutrition	Adults	84.5 (11)	87.2 (24)	0.84	0.15	0.97
	Adolescents	83.3 (18,7)	92 (17)			

^1^ *p* < 0.05, pre versus post.
